# The EVERT (Effective Verruca Treatments) Trial: a randomised controlled trial to evaluate cryotherapy versus salicylic acid for the treatment of verrucae

**DOI:** 10.1186/1757-1146-3-S1-O6

**Published:** 2010-12-20

**Authors:** Sarah Cockayne, David Torgerson, Mike Curran, Nichola McLarnon, Kim Thomas, Farina Hashmi

**Affiliations:** 1 University of York, York, UK; 2 University of Northampton, Northampton, UK; 3 Glasgow Caledonian University, Glasgow, UK; 4 University of Nottingham, Nottingham, UK; 5 University of Brighton, Brighton, UK

## Objectives and relationship to conference themes

Verrucae are a common, infectious and sometimes painful problem. The optimal treatment for verrucae is unclear due to a lack of high quality randomised controlled trials (RCTs). The primary objective of the EVERT trial is to compare the clinical effectiveness of two common treatments for verrucae: cryotherapy using liquid nitrogen delivered by a healthcare professional versus patient self-treatment with salicylic acid. We aim to disseminate the trial results amongst podiatrists and other healthcare professionals who treat verrucae, via presentation at the SCP Annual Conference 2010 and publication in a peer-reviewed journal.

## Content of presentation

The oral presentation will include background to the EVERT trial, including previous relevant research and the need for the current trial, trial design, the results of the trial and the conclusions drawn from the study.

## Relevance/impact of topic

A systematic review conducted by the Cochrane Skin Group (BMJ, 2002) highlighted the uncertainty with respect to the optimal treatment of verrucae. Only two trials were identified which compared salicylic acid and/or lactic acid with cryotherapy, and they found no difference in the efficacy between the treatments. However, both trials were reported as low quality, hence the current need for a high quality RCT with a cost effectiveness analysis to ascertain which is the better approach. The results of the EVERT trial will be used to inform clinical practice.

## Participant outcomes

The statistical and economic analyses will be completed by July 2010. The primary outcome is complete clearance of all verrucae at 12 weeks. Secondary outcomes are time to clearance of verrucae, clearance of verrucae at six months, pain intensity of the first treatment, use of painkillers due to verruca treatment, and patient satisfaction with the treatment. The primary economic evaluation will be a cost-effectiveness analysis of the trial treatments.

